# Understanding of the characteristics of fibroblasts in ischemic cardiomyopathy using single-nucleus RNA sequencing

**DOI:** 10.1038/s41598-025-00260-7

**Published:** 2025-05-30

**Authors:** Qianyuan Zhang, Ziwen Cai, Yongqiao Zhang

**Affiliations:** 1 Department of Cardiovascular Surgery, Fujian Medical University Union Hospital, Fuzhou, 350001 Fujian China; 2 Key Laboratory of Cardio-Thoracic Surgery, Fujian Medical University, Fujian Province University, Fuzhou, Fujian China; 3 Fujian Provincial Center for Cardiovascular Medicine, Fuzhou, Fujian China; 4 Department of General Medicine, Fujian Medical University Union Hospital, Fuzhou, Fujian China

**Keywords:** Ischemic cardiomyopathy, Coronary artery disease, Single-nucleus RNA sequencing, Transcriptome, Fibroblasts, Cardiology, Molecular medicine, Pathogenesis

## Abstract

Fibroblasts play a crucial role in the development and progression of heart failure in many heart-related diseases. However, the contributions of fibroblast and its subtypes to the pathogenesis of ischemic cardiomyopathy (ICM) remain unclear. In this study, we analyzed single-nucleus RNA sequencing data from cardiac tissues of seven ICM patients with heart failure and eight control subjects. Our findings indicate that, compared to controls, fibroblasts from ICM patients exhibit dysregulation of genes associated with cell-substrate adhesion and matrix or collagen fibril organization. We further identified five fibroblast subtypes, designated as FB1 through FB5, with FB3 exhibiting the highest frequency in ICM. Pseudotime trajectory analysis further revealed an evolutionary progression of fibroblasts from FB1/FB2 toward FB3. Differential gene expression analysis of FB3 highlighted several subtype-specific genes, such as NTM, THBS4, NRK, NAV2, and PLA2G5. Furthermore, intercellular interaction analysis revealed that FB3 exhibits weakened interactions with other cells under ICM. Specifically, signaling interactions involving ANGPTL and COLLAGEN are reduced, whereas pathways mediated by THBS, NRXN, and APP are strengthened. These results provide valuable insights into the fibroblast microenvironment and elucidate the pathological mechanisms underlying ICM, laying a theoretical foundation for the identification of novel therapeutic targets.

**Supplementary Information:**

The online version contains supplementary material available at 10.1038/s41598-025-00260-7.

## Introduction

Ischemic cardiomyopathy (ICM), defined as systolic left ventricular dysfunction in the context of obstructive coronary artery disease (CAD), is a primary cause of heart failure (HF)^1^. HF represents a severe cardiac dysfunction with high activity levels and mortality rates. Compared to non-ischemic HF patients, those with CAD-induced HF exhibit worse prognoses and higher mortality rates^2,3^. Although advancements in medical technology have improved disease progression through various clinical interventions-including medication, thrombolysis, stent implantation, and coronary artery bypass surgery-no curative treatment exists. For patients with advanced HF, the one-year survival rate remains only around 50%, posing a serious threat to patient health^4,5^. Therefore, understanding the pathophysiological mechanisms underlying ICM is essential for developing novel therapeutic and diagnostic approaches.

Cardiac fibroblasts, scattered in striated and sheet-like forms throughout the heart, are the main cell types responsible for extracellular matrix (ECM) deposition. These cells provide structural support for contracting cardiomyocytes and participate in diverse physiological signaling processes, including ECM remodeling, myofibroblast differentiation, and cardiomyocyte regulation^6,7^. In ischemic cardiomyopathy, fibroblasts are activated and differentiate into myofibroblasts, which are responsible for the deposition of extracellular matrix components. This process forms fibrotic scars that replace necrotic tissue, preventing myocardial rupture and promoting wound healing and repair. However, excessive proliferation and activation of fibroblasts can lead to excessive fibrotic scarring and functional impairment, thereby contributing to the development of heart failure and arrhythmias^8^. Badder Kattih has confirmed persistent fibroblast activation in chronic heart disease^9^. Additionally, immune cells, vascular cells, and cardiomyocytes may also acquire a fibrotic phenotype under stress conditions, further promoting fibroblast activation^10^. However, there are currently limited clinical interventions specifically targeting cardiac fibroblasts and their role in fibrotic tissue deposition. Therefore, a more detailed characterization of cell-specific changes is needed to guide the development of novel diagnostic and therapeutic targets.

Single-cell RNA sequencing (scRNA-seq) enables high-throughput measurement of gene expression in thousands of individual cells simultaneously. It has emerged as a state-of-the-art method for elucidating the heterogeneity and complexity of RNA transcripts within single cells. This technique can reveal the composition of different cell types and their functions in highly organized tissues, thereby deepening our understanding of disease pathogenesis and providing valuable insights for the development of novel diagnostic and therapeutic strategies^11,12^. In this study, we utilized single-cell analysis tools to comprehensively analyze publicly available adult cardiac snRNA-seq datasets, aiming to investigate fibroblast-specific transcriptomic alterations associated with end-stage ICM and reveal the pathological mechanisms involved in disease progression.

## Materials and methods

### Data collection

The data of this study is obtained from the public snRNA data in Broad Institute’s Single Cell Portal (SCP1849)^13^. In brief, the processed expression matrixes from in total of 15 samples, which included 8 healthy (named as Normal group) and 7 ICM individuals (named as ICM group) were downloaded. We further selected the good-quality nuclei by the following standards: the detected gene number in the range from 200 to 8000, UMI counts greater than 500, and mitochondrial ratio less than 5%. Finally, a total of 80,315 nuclei were kept for further analysis.

### Cell clustering and cell type identification

We mainly used R software (version 4.2.1) and the Seurat library (version 4.3.0.1) to process the snRNA data. The SCTransform function in Seurat was utilized to perform UMI count NFization, top 2000 high variable genes (HVGs) identification, and expression data scaling for each sample simultaneously. Next, all the processed single sample data were integrated with RunHarmony function to eliminate the batch effects among multiple samples. The top 3000 integrated HVGs selected from the samples were used to perform principal components analysis and dimension reduction. Finally, FindNeighbors and FindClusters function were jointly used to cluster the cells in an unsupervised style. We set the resolution value to 0.8 for cell clustering. The distribution of cell clusters was shown by UMAP (Uniform Manifold Approximation and Projection) plot by the RunUMAP command. To identify the cell types for each cell cluster, we combined the information of the expression known markers for the main cell types in the human heart and the specific highly expression genes in each cluster found by FindAllMarkers.

### Subtype clustering of fibroblasts

Fibroblasts were selected based on the cluster with the specific expression of DCN and CDH19. Following the cell-clustering procedures of the major cells, the fibroblasts were also processed with HVGs identification and data integration, and then were divided into several clusters with a resolution of 0.6. To reveal the heterogeneity and characterizations of sub-fibroblasts, the mark genes of each cluster were identified using FindAllMarkers.

### Activation score computation of fibroblast subtypes

We used AddModuleScore function in Seurat library to infer activation scores of different fibroblast subtypes. The gene signatures were selected based on the previous report^14^, which included POSTN, THBS4, TSHZ, FAM155, COL1 A2, MIR100HG, NOX4, COL22 A1, FAP and COL1 A1.

### Trajectory analysis of subtypes of fibroblasts

The diversity of fibroblast subtypes indices the states and functions transferring. To reveal the relationships, we performed the trajectory analysis of the subtypes using monocle library (version 2.20.0)^15^. The order genes among different fibroblast subtypes were selected using function differentialGeneTest. The cellular trajectory was constructed by functions reduceDimension and orderCells. The BEAM function was used to perform trajectory branch analysis, and the plot_genes_branched_heatmap was used to visualize the top trended genes along with different branches.

### Differential expression analysis

To identification the differential expression genes (DEGs) between Normal and ICM groups, we performed differential expression analysis among the fibroblasts or the different fibroblast subtypes using FindMarkers function in the Seurat library, and wilcoxon rank sum test was used. Following that, GO annotation and KEGG pathway^16^analysis for DEGs were performed by clusterProfiler library (version 4.0.5)^17^.

### Cell-cell interactions between Fib subtypes with other cells

We used CellChat library (version 1.6.1)^18^ to analyze the cell-cell interactions between FB3 and other immune and no-immune cells. The normalized expression data were input into CellChat to create the object. Then the expression levels of receptors and ligands were assessed among the cell groups. The ligand-receptor interactions recorded in the CellChatDB.human database utilized in this study can be categorized into three categories: secrete autocrine/paracrine signaling interactions, extracellular matrix-receptor interactions and cell-cell contact interactions.

### External data validation

To validate fibroblast subtype-specific signatures, we analyzed bulk RNA-seq data from GSE116250^19^13 ICM vs. 13 healthy left ventricles) using DESeq2 (v1.32.0), defining differential genes (ICM vs. controls) as |log2 FC| > 1.5 and qvalue (pvalue adjust) < 0.05. Proteomic validation utilized iProX dataset (ID: IPX0005712000^20^, including 4 ICM and 3 controls) processed with R DEP (v1.28.0) package, differentially expressed proteins with fold change > 1.5 and *p* < 0.05. Functional enrichment analysis of differentially expressed molecules was performed using the R package clusterProfiler (v4.0.5). Significance was determined via the hypergeometric test with subsequent Benjamini-Hochberg (BH) multiple testing correction, retaining terms with adjusted p-values (FDR) < 0.05. Protein-protein interaction (PPI) networks among differentially expressed proteins were constructed using Metascape and visualized in Cytoscape (v3.4.0). Key candidate genes were visualized as violin plots using ggplot2 (v3.5.1) to compare their expression distributions between ICM patients and healthy controls.

## Results

### Identification of cell populations with ischemic cardiomyopathy

**Fig. 1:**
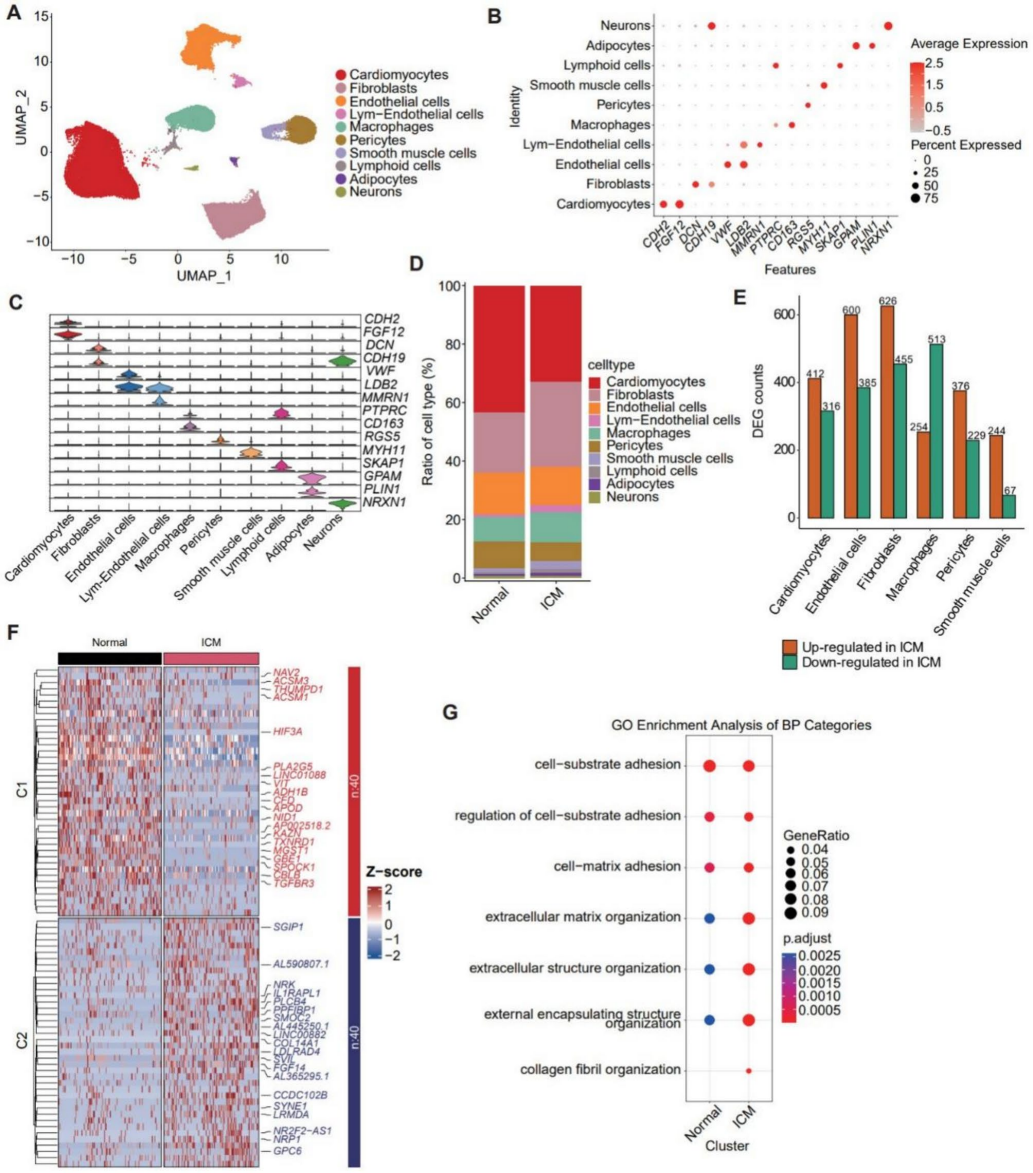
Cellular Landscapes of the human heart from Normal and ICM patients revealed by snRNA sequencing. (**A**) UMAP plot showing the ten main cell types in the human hearts. (**B**) Dot plots showing the expression levels of the markers for cell types. (**C**) Violin plots the expression levels of the markers for each cell type. (**D**) Bar plots showing the cell type frequency distribution between Normal and ICM groups. (**E**) The up-regulated and down-regulated gene counts of ICM compared to Normal group for each main cell type separately. (**F**) Heatmap plot showing the differential expressed genes (DEGs) in fibroblasts between Normal and ICM groups. The top 20 up-regulated and down-regulated genes were labeled. (**G**) GO Enrichment analysis of the DEGs in fibroblasts between Normal and ICM groups.

To identify and characterize changes associated with the progression of ICM to heart failure, we utilized a scRNA-seq dataset from a prior study that examined the non-infarcted regions of the left ventricle (LV) in patients with long-term ICM (*n* = 7) and healthy controls(*n*= 8)^13^. The mean age of the two groups was 58 ± 6.2 years and 54.63 ± 7.6 years, respectively, and the mean BMI was 29.9 ± 4.5 and 30.2 ± 8.4, respectively. The ICM group consisted of 2 males and 5 females, while the control group included 4 males and 4 females. Among the ICM group, 5 patients had hypertension, and 3 patients had diabetes, while in the NF control group, 2 patients had hypertension. There were no statistically significant differences between the two groups in terms of age, gender, BMI, and comorbidities. After filtering, a total of 80,315 high-quality nuclei were retained for further analysis. Using unsupervised clustering, the cells were grouped into 21 clusters (Figure S1A). Most clusters displayed comparable values for key metrics such as Feature_RNA, nCount_RNA, and percent.mt (Figure S1B). Based on gene expression patterns associated with different cell types, these clusters were classified into ten major cell types, including cardiomyocytes, fibroblasts, endothelial cells, lymphatic endothelial cells, macrophages, pericytes, smooth muscle cells, lymphoid cells, adipocytes, and neurons (Fig. 1A and C). The cell clustering analysis revealed an even distribution of cells from both the ICM and control groups across these cell types, indicating that potential batch effects were successfully mitigated during data processing (Figure S1C–S1D). We further compared the proportional distribution of each major cell type between the two groups, finding that cardiomyocytes were the most abundant cell type, followed by fibroblasts and endothelial cells (Fig. 1D). Differential gene expression analysis between the ICM and control groups revealed that fibroblasts had the highest number of differentially expressed genes, with 626 upregulated and 455 downregulated genes (Fig. 1E). A heatmap of the top 20 upregulated and downregulated genes in fibroblasts showed notable upregulated genes such as HIF3 A, NAV2, SGIP1, MGST1, and TXNRD1, and downregulated genes including COL14 A1, VIT, ADH1B, APOD, and GPC6 (Fig. 1F). Pathway enrichment analysis indicated associations with cell-substrate adhesion and matrix or collagen fibril organization, which is related to ECM production and remodeling, cell proliferation and migration (Fig. 1G).

### The subcluster heterogeneity of fibroblasts

**Fig. 2:**
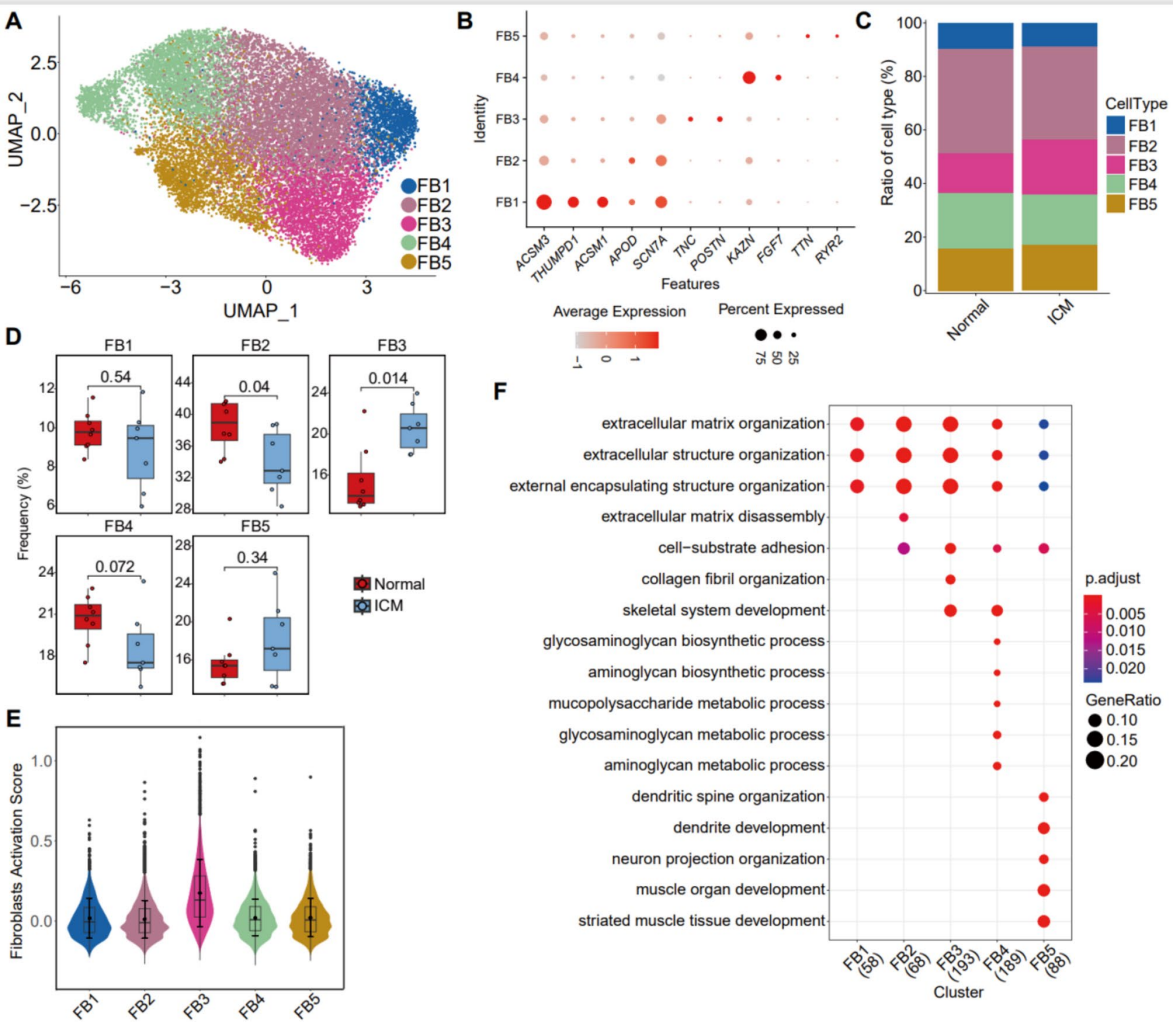
The subcluster heterogeneity of fibroblasts. (**A**) UMAP plot showing the fibroblast subtypes: FB1-FB5. (**B**) Dot plot showing the marker genes across all the fibroblast subtypes. (**C**) Bar plots showing the frequency distribution of fibroblast subtypes between Normal and ICM groups. (**D**) Box plot showing the statistics comparison of the abundance of FB1 to FB5 between Normal and ICM groups. The Wilcoxon rank-sum test method is used for comparison. (**E**) Violin plots showing the fibroblast activation score across the fibroblast subtypes. (**F**) GO terms for biological processes (BP) ontology enriched for the highly expressed genes in each subtype.

To further investigate fibroblast heterogeneity, we isolated fibroblasts for re-clustering analysis. A total of 19,028 fibroblasts from both Normal and ICM groups were categorized into ten clusters (Figure S2A-C). Based on gene expression characteristics, these clusters were annotated into five distinct subtypes, labeled FB1-FB5 (Fig. 2A). FB1 was characterized by high expression of ASCM3, THUMPD1, ASCM1, and SCN7 A. FB2 showed overlap in marker genes with FB1, such as APOD and SCN7 A, while the expression levels are weaker than FB1. FB3 was defined by high expression of TNC and POSTN, FB4 by specific high expression of KNZN and FGF7, and FB5 by markers TTN and RYR2 (Fig. 2B). Cell proportion analysis revealed variation in the abundance of FB subtypes across samples, with FB2 displaying a decrease in the ICM group, while FB3 showed a significant increase in proportion in the ICM group (*p* = 0.014) (Fig. 2C-D). Moreover, gene signature scoring analysis revealed that FB3 exhibited the highest fibroblast activation activity (Fig. 2E), which may contribute to the progression of ICM by promoting fibrosis. GO enrichment analysis indicated that all FB subtypes (FB1–FB5) were enriched in biological processes related to ECM formation and structure. Further GO analysis based on molecular function and structure highlighted that FB1 was associated with enzymatic activities and transporter activity. FB2-FB4 were related to ECM interactions, with FB2 also linked to ion channel and transporter activity, FB3 to cytoskeletal and cell adhesion molecule interactions, FB4 to receptor and signal transduction activity, and FB5 to regulator activity. Additionally, FB4 was enriched in glycosaminoglycan and aminoglycan metabolism pathways, while FB5 was enriched in pathways associated with neural development and organization (Fig. 2F & S2D-E).

### Differentiation trajectory of fibroblast subtypes

**Fig. 3:**
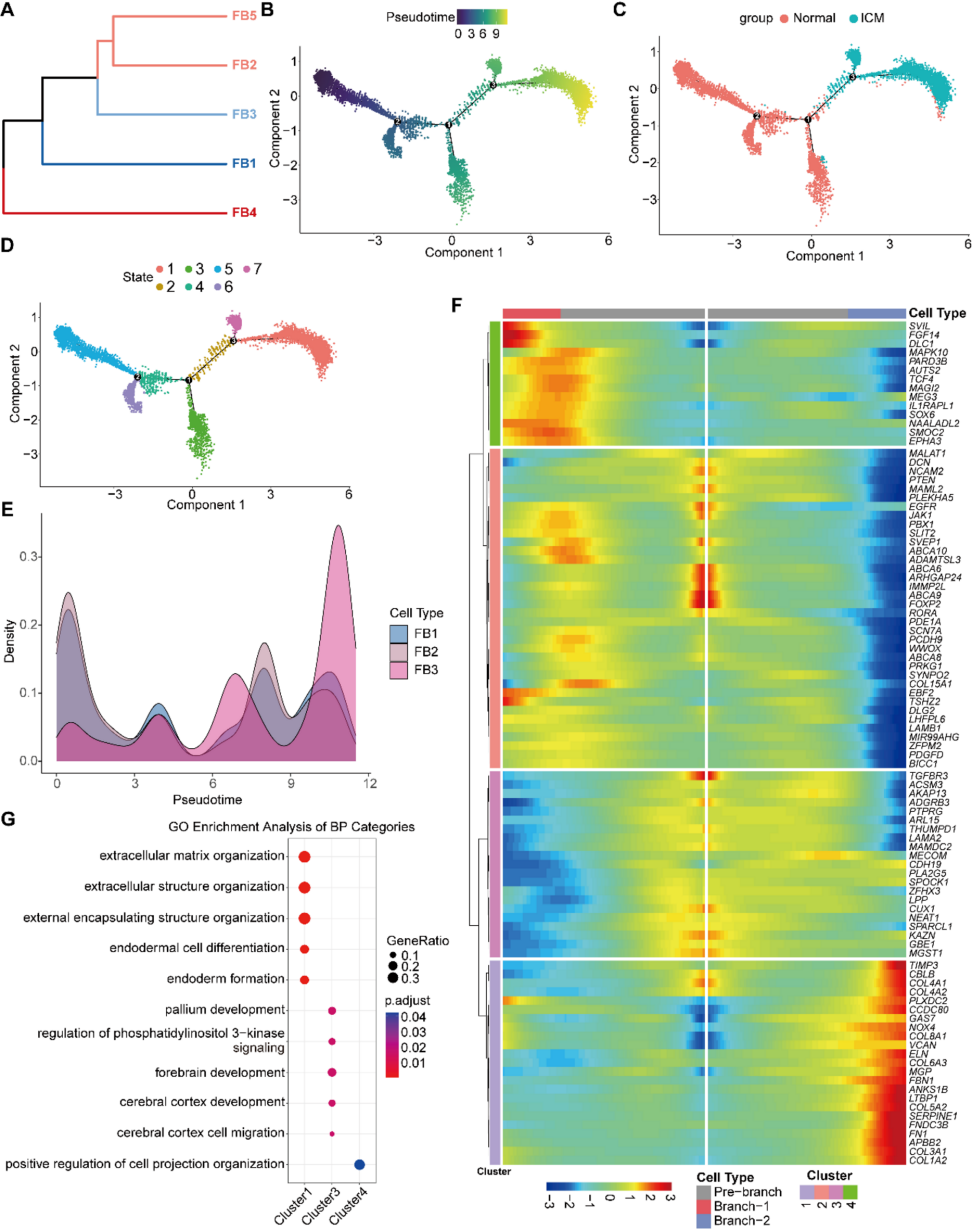
Differentiation trajectory of fibroblast subtypes. (**A**) Phylogenetic Tree constructed based on the gene expression profiles of the subtypes of fibroblast. (**B**) Pseudotime trajectory of cells from FB1, FB2 and FB3, and smaller pseudotime values mean earlier differentiation stages. (**C**) Cellular trajectory colored by different original groups. (**D**) Cellular trajectory colored by 7 different states, and can be divided into 3 branches, with State4,5,6 as Pre-Branch, State1,7 as Branch-1, and State3 as Branch-2. (**E**) The density distribution plot of FB1, FB2 and FB3 along with pseudotime values. (**F**) BEAM heatmap plot showing the differential expression genes along with Pre-Branch, Branch-1 and Branch-2 stages in the differentiation trajectory. (**G**) GO Enrichment analysis of the four differential expressed gene clusters (Cluster1-4) along with the differentiation branches. The results focused on the biological processes (BP) ontology, and there were no enriched terms from Cluster2 gene set.

To further elucidate the evolutionary relationships among different fibroblast subtypes, we first employed clustering analysis to construct a phylogenetic tree by dendextend library (version 1.15.2). This analysis revealed that FB2, FB5, and FB3 share the closest phylogenetic relationship, followed by FB1, while FB4 emerges as a separate branch, indicating distinct gene expression profiles and differentiation characteristics specific to FB4 (Fig. 3A). Subsequently, pseudotime analysis was utilized to investigate the differentiation relationships among subtypes, excluding FB4. It was found that FB3 and FB5 have unique terminal differentiation characteristics but share a common origin in FB1/FB2 (Figure S3A). To further characterize the evolutionary trajectory and driver genes of FB3, we conducted a specific analysis of cells from FB1 to FB3. The differentiation trajectory revealed two distinct differentiation branches among the three FB subtypes (Fig. 3B). The cell trajectory could be divided into seven states, with States 3/4/5/6 primarily originating from normal fibroblasts, while States 1/7 mainly derived from ICM fibroblasts (Fig. 3C-D). This suggests significant divergence in differentiation trajectories between normal and ICM fibroblast subtypes. Among cell types, FB3 demonstrated a greater pseudotime value than FB1 and FB2, indicating a differentiation sequence from FB1/FB2 to FB3. Furthermore, there were notable differences in the differentiation paths of FB3 originating from normal versus ICM sources, highlighting the unique characteristics of FB3 under ICM conditions (Fig. 3E &S3B). Based on node distribution, States 4, 5, and 6 were designated as Pre-Branch, States 1 and 7 as Branch-1, and State 3 as Branch-2. BEAM analysis was then used to identify driver genes at different differentiation stages. The results showed stage-specific expression of driver genes, with some genes being up-regulated and others down-regulated, reflecting the biological behaviors of cells under various developmental or pathological conditions. In particular, genes in Cluster 4, such as SVIL, FGF14, and DLC1, were up-regulated in Branch-1, whereas genes in Cluster 3, like ADGRB3, CDH19, MECOM, and PLA2G5, were down-regulated. Additionally, genes in Cluster 1 were upregulated in Branch-2, including FN1, COL1 A2, TIM3, and COL3 A1, while genes in Cluster 2/4, such as MAPK10, AUTS2, DCN, and PTEN, were down-regulated in Branch-2 (Fig. 3F). Pathway enrichment analysis indicated that Cluster 1 is associated with extracellular matrix or structure organization, endodermal cell differentiation and formation. Cluster 3 is associated with cerebral cortex matrix, pallium, forebrain development or migration, and regulation of phosphatidylinositol 3-kinase signaling (Fig. 3G).

### Differential gene expression analysis between normal and ICM groups across fibroblast subtypes

**Fig. 4:**
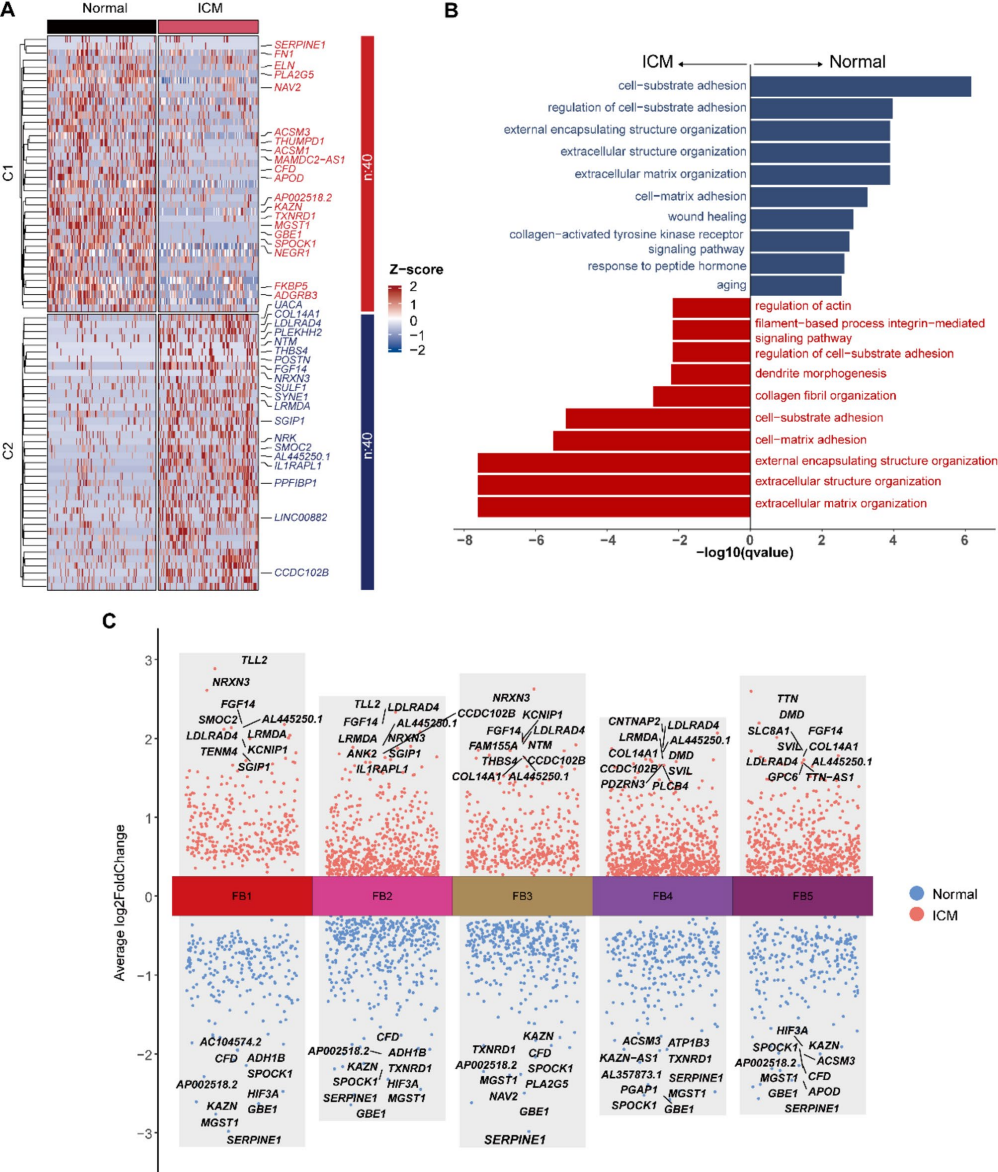
Differential gene expression analysis between Normal and ICM groups across fibroblast subtypes. (**A**) Heatmap plot showing the differential expressed genes (DEGs) in FB3 between Normal and ICM groups. The top 15 up-regulated and 15 down-regulated genes were labeled. (**B**) Bar plot showing the enriched GO BP terms of ICM-upregulated and ICM-downregulated genes. (**C**) Volcano map showing the DEGs across the five subtypes of fibroblast. Blue means the up-expressed genes in Normal, and red means the up-expressed genes in ICM. The top 5 up or down-expressed genes were labeled.

In our analysis of differentially expressed genes between normal and ICM conditions across fibroblast subtypes. Based on the log2 FC value, we identified the top five upregulated genes in FB3 as NRXN3, KCNIP1, FGF14, LDLRAD4, and FAM155 A, and the top five downregulated genes as SERPINE1, GBE1, NAV2, MGST1, and PLA2G5 (Fig. 4A, Supplementary Table 1). Gene Ontology enrichment analysis revealed shared pathways between upregulated and downregulated genes, including extracellular matrix organization, extracellular structure organization, and external encapsulating structure organization pathways (Supplementary Table 2). Specific to downregulated genes, the wound healing pathway was identified, which may relate to tissue repair processes^3^. In contrast, the upregulated genes in ICM conditions highlighted the collagen fibril organization pathway, previously reported to be associated with fibrotic processes in hypertrophic cardiomyopathy^4^ (Fig. 4B). And we obtained similar findings through functional validation using public databases (Figure S5&S6). Bulk RNA-seq data from the GSE116250 cohort revealed significant overlap between ischemic cardiomyopathy (ICM)-associated differentially expressed genes (DEGs) and FB3-specific markers (THBS4, NTM, NRK, COL14 A1 upregulated; MGST1 downregulated) (Figure S5A, C, Supplementary Table 3). Functional enrichment of overlapping DEGs highlighted extracellular matrix (ECM)-related processes such as “collagen fibril organization” (FDR < 0.05; Figure S5B), mirroring our single-cell findings. Proteomic validation using the IPX0005712000 dataset further corroborated these results. Upregulated proteins in ICM tissues were enriched in pathways critical to fibroblast activation, including “supramolecular fiber organization” and “actin cytoskeleton regulation” (Figure S6A, B, Supplementary Table 4). Notably, FB3-associated proteins SVIL, MYO1D, and PLS3 exhibited significant overexpression in ICM samples (*p* < 0.01; Figure S6C), consistent with their elevated expression in FB3-derived snRNA-seq data (Supplementary Table 1). The concordance across transcriptomic, proteomic, and single-cell layers underscores the robustness of FB3 as a fibrosis-driving subpopulation in ICM pathogenesis. Detailed validation results are provided in Supplementary Data.

Subsequently, we compared the gene expression in FB3 relative to other FB subtypes (Fig. 4C& S4 A-C), identifying FB3-specific upregulated genes, including FAM155 A, NTM, THBS4, and NRK. Shared upregulated genes across subtypes included LDLRAD4 and the lncRNA AL445250.1. Among the downregulated genes, FB3-specific genes were NAV2 and PLA2G5, while shared genes included SERPINE1, MGST1, GBE1, and SPOCK1.

### Cell-cell interactions between FB3 and other cell types

**Fig. 5:**
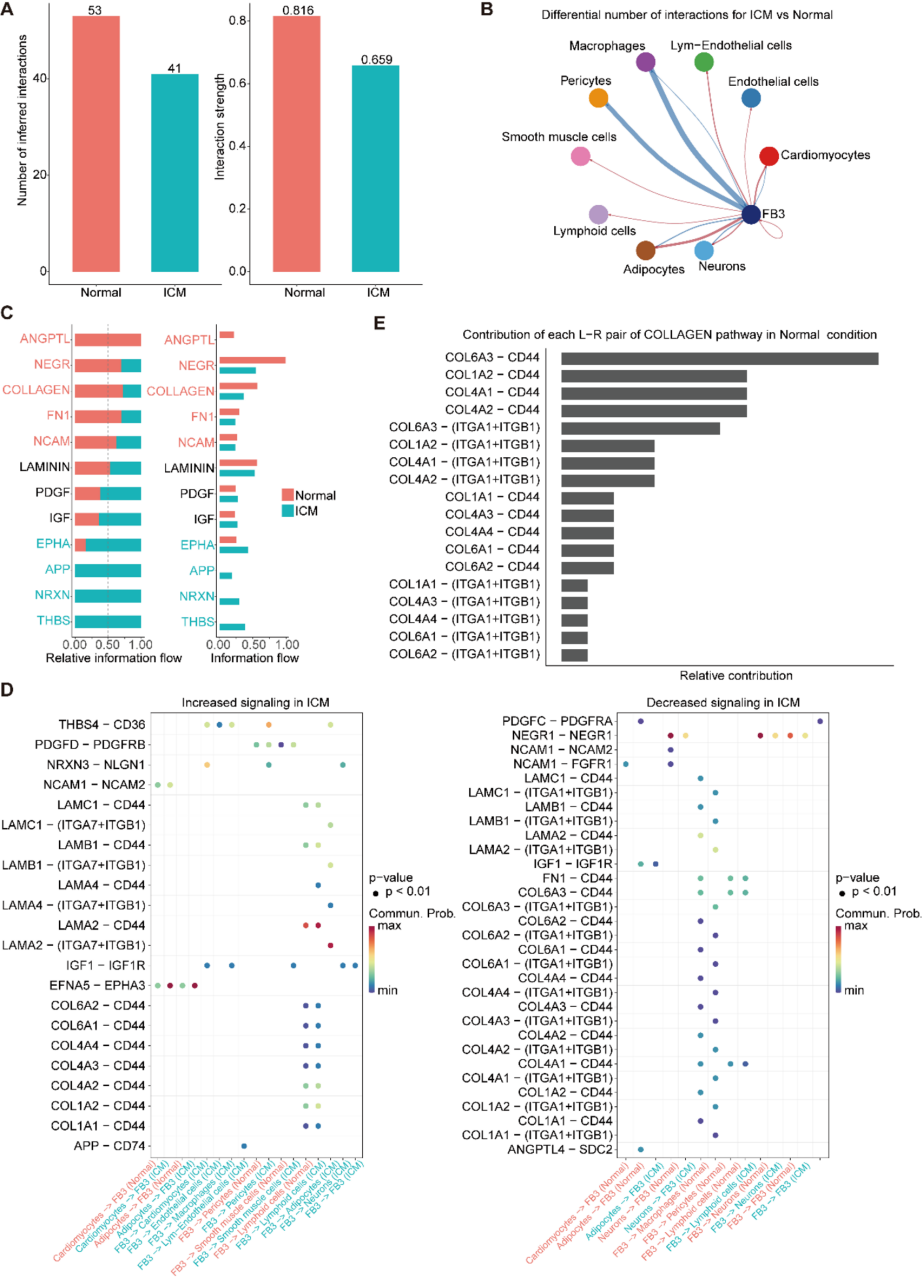
Cell-cell interactions between FB3 and other cell types. (**A**) Bar plot showing the inferred interaction number (left) and interaction strength (right) between FB3 and other cell types in Normal and ICM groups. (**B**) Network plot showing the differential interaction strength between FB3 with other cell types. Blue linkers mean the interaction is weaker in ICM, and red linkers mean the interaction is stronger in ICM compared to Normal. (**C**) The information flow comparison of interaction strength between two groups, the results are shown in stacked style (left) or not (right). The pathways present stronger in Normal are colored red, and those stronger in ICM are colored blue. (**D**) Bubble plotting of the ligand-receptor pairs with increased (left) and decreased (right) interaction strength among the cell types in ICM compared to Normal. (**F**) COLLAGEN pathway of ligand-receptor pairs.

We utilized CellChat to assess the interactions of FB3 with other cells and compared the differences between the interactions under ICM and normal conditions. Overall, under ICM conditions, both the number and strength of interactions between FB3 and other cells were reduced (Fig. 5A). Network analysis revealed that this reduction was primarily due to weakened interactions with macrophages and pericytes; however, interactions with cardiomyocytes and adipocytes were enhanced (Fig. 5B). Further, we conducted an information flow analysis to compare the changes in the quantity and strength of signaling pathway interactions. Compared to the normal group, the ICM group exhibited reduced interactions involving the ANGPTL and COLLAGEN pathways, whereas interactions in the THBS, NRXN, and APP pathways were strengthened (Fig. 5C). Subsequently, we evaluated the ligand-receptor pairs involved in these signaling pathways across different cell types (Fig. 5D). Among the increased signaling in ICM, THBS pathway (THBS4-CD36 pair) was ICM specified, and sourced from FB3 and targeted to cardiomyocytes, endothelial cells, Lym-Endothelial cells, pericytes and adipocytes. NRXN pathway (NRXN3-NLGN1 pair) also was ICM specified and mainly from FB3 to cardiomyocytes. APP pathway (APP-CD74 pair) also sourced from FB3 and taken macrophages as target. When insight into the decreased signaling in ICM, we found COLLAGEN pathway engages multiple ligand-receptor pairs (Fig. 5E), and much of these ligand-receptor pairs present decreased interaction strength, and these interactions mainly sourced from FB3 to macrophages or pericytes.

## Discussion

ICM results primarily from chronic coronary insufficiency, which causes extensive myocardial dysfunction and structural changes. Clinically, it manifests as symptoms like dyspnea and chest pain, and in advanced stages, can lead to heart failure, severe arrhythmias, and even death^7,21^. Therefore, there is an urgent need to clarify the pathophysiological mechanisms underlying ICM and identify meaningful diagnostic and therapeutic targets.

Cardiac fibroblasts (CFs) play a central role in synthesizing and degrading the ECM. Cardiac fibrosis, characterized by excessive ECM deposition, is a key pathological mechanism in heart failure and often occurs in conditions of cardiac injury, inflammation, or aging^22^. In this study, we identified dysregulated gene expression in CFs associated with biological processes such as inflammation, wound healing, cellular proliferation, migration, and oxidative responses in ICM. Specifically, genes like NAV2, SPOCK1, TGFBR3, APOD, and PLA2G5 were upregulated, while others, including NRK, GPC6, and FGF14, were downregulated. Previous studies have reported similar findings, but most have focused on differential gene expression in tumor cells or non-cardiac fibroblasts. For example, NAV2 and SPOCK1 upregulation is known to induce epithelial-mesenchymal transition (EMT) and promote tumor migration and invasion^23,24^or immune infiltration in colorectal cancer^25^. Furthermore, PLA2G5 and PLA2G2, which are associated with inflammation and fibrosis-related pathways, show increased expression in fibroblasts from patients with idiopathic pulmonary fibrosis, thus activating TGF-β, IL-17 signaling pathways, arachidonic acid metabolism, and ECM-receptor interactions^26^. Moreover, NRK upregulation has been documented in benign prostatic hyperplasia samples; NRK downregulation inhibits stromal cell proliferation, migration, fibrosis, and EMT processes, promoting apoptosis and inducing cell cycle arrest^27^. Our findings enhance understanding of the role of cardiac fibroblasts in advanced ICM and highlights potential targets for therapeutic intervention.

Further analysis of CF subtypes revealed five FB subgroups (FB1-FB5), each enriched in ECM-related biological pathways. We observed a differentiation trajectory from FB1/FB2 to FB3, with distinct patterns between normal and ICM-derived fibroblasts. FB1 and FB2 exhibited differentiation patterns similar to those in normal hearts, whereas FB3 aligned more closely with the ICM state. Genes in Clusters 2 and 4, such as FN1, COL family members, DCN, JAK1, PTEN, TIMP3, and MALT1, are potential drivers of FB2 differentiation. FN1, COL1 A2, COL3 A1, and DCN are key ECM components, involved in ECM production and remodeling^28^.TIMP3^29^, PTEN^30^, JAK1^31^, and MALT1^32,33^ are involved in immune signaling. These findings suggest that the FB2 subtype may represent an early fibroblast phenotype, with driver genes mainly involved in initial tissue repair and responses.

In contrast, genes in Clusters 1 and 3—such as SVIL, SPOCK1, FGF14, DLC1, and PLA2G5—are likely contributors to FB3 differentiation. SVIL and SPOCK1 participate in cytoskeletal remodeling and may promote fibroblast migration and proliferation, potentially facilitating pathological changes^34^. For instance, SVIL promotes ovarian cancer progression and EMT under hypoxic conditions via the TGF-β/Smad pathway^35^. FGF14 regulates voltage-gated sodium channels^36^, while DLC1 is linked to atrial pacing and intercellular signaling, with loss-of-function mutations potentially leading to arrhythmias^37^. PLA2G5 is implicated in blood pressure homeostasis, inflammation, and metabolic gene expression^38^. These findings suggest that FB3 represents a more mature or pathologically fibrotic subtype that drives fibrosis progression. Moreover, the current literature on single-cell sequencing studies using transplantation samples is limited. Research has primarily addressed cellular landscapes in end-stage heart failure due to conditions such as valvular heart disease^39^, arrhythmogenic right ventricular cardiomyopathy^40^, functional impairment of perivascular adipose tissue (PVAT)^41^, and dilated cardiomyopathy^42^. Notably, the study on ICM has predominantly focused on the subtyping of endothelial cells, and subtype analysis of fibroblasts in heart failure due to ICM has not been previously reported. Therefore, this study provides new evidence for the potential pathological mechanisms involved in advanced ICM.

Further interaction analysis of FB3 with other cell types revealed weakened interactions with macrophages and pericytes, while its interactions with cardiomyocytes and adipocytes were enhanced. As ICM progresses, pericytes can differentiate into myofibroblasts, leading to microvascular degeneration, ECM accumulation, and tissue stiffening^43,44^. This progression possibly reduces their function as pericytes, thereby weakening their interaction with FB3. Immune cells, both resident and recruited, play an essential role in cardiac injury by modulating inflammatory cell recruitment and activation through interaction with fibroblasts. In DCM, enhanced fibroblast–macrophage interaction has been observed, particularly in the early stages of the disease^42,45^. However, our findings show that this interaction is weakened in late-stage ICM. This may be due to the functional decline of immune cells following prolonged or excessive activation in advanced disease stages. For instance, neutrophil depletion can exacerbate cardiac fibrosis and induce HF^46^. Future studies are warranted to further explore this hypothesis. Additionally, damaged cardiomyocytes or adipocytes may release specific signaling molecules that activate FB3, promoting its proliferation and differentiation or altering the cardiac metabolic state. This, in turn, may increase fibrin deposition, contributing to fibrosis or cardiac remodeling^9,47–49^. Notably, analysis of interaction pathways revealed that the THBS, NRXN, and APP pathways are more active in the ICM group. THBS4, an adhesive glycoprotein, mediates cell–cell and cell–matrix interactions^50,51^.Overexpression of THBS4 has been reported to induce fatal cardiac atrophy^52^. These results further support the role of FB3 in promoting pathological fibrosis through intercellular interactions in ICM.

While our study provides multi-omics insights into fibroblast heterogeneity in ischemic cardiomyopathy (ICM), several limitations should be acknowledged. First, the sample size of snRNA-seq cohort (7 ICM vs. 8 controls) and proteomic validation dataset (4 ICM vs. 3 controls) is relatively limited, which may introduce variability in identifying rare fibroblast subtypes or subtle molecular shifts. Although we mitigated this by cross-validating subtype-specific signatures (e.g., THBS4, SVIL) against bulk transcriptomic data and independent proteomic profiles, larger cohorts are needed to enhance the generalizability of our findings. Second, while our integrative analysis revealed concordant dysregulation of ECM remodeling and actin cytoskeleton pathways across transcriptomic and proteomic layers, functional validation (e.g., CRISPR-based knockdown of THBS4 in fibroblast cultures or in vivo models) remains to be performed. Such experiments are critical to establish causal relationships between FB3 activation and ICM progression. In future studies, we plan to expand our cohort with additional ICM samples and incorporate spatial transcriptomics to resolve fibroblast localization within fibrotic niches. Mechanistic validation of prioritized targets (e.g., THBS4-mediated collagen deposition) will be pursued using patient-derived cardiac fibroblasts and preclinical models. Despite these limitations, our multi-omics framework establishes FB3 as a key mediator of pathological fibrosis and provides a roadmap for discovering therapeutic strategies targeting fibroblast plasticity in ICM.

## Conclusions

In summary, this study used single-cell RNA sequencing to reveal common and unique gene expression patterns in fibroblasts from normal and ICM heart tissues, including disrupted signaling pathways, cellular developmental trajectories, and intercellular crosstalk involving FB3 and other cell types. Our data provide a valuable resource for deepening the understanding of mechanisms associated with ICM progression and may aid in the development of more effective therapeutic targets.

## Electronic supplementary material

Below is the link to the electronic supplementary material.

Supplementary Material 1

Supplementary Material 2

Supplementary Material 3

Supplementary Material 4

Supplementary Material 5

Supplementary Material 6

Supplementary Material 7

Supplementary Material 8

## Data Availability

All the data in this study is public, and obtained from the Broad Institute’s Single Cell Portal: https://singlecell.broadinstitute.org/single_cell/study/SCP1849/.

## Abbreviations

ICM: Ischemic cardiomyopathy
snRNA-seq: Single-nucleus RNA sequencing
HF: Heart failure
CAD: Coronary artery disease
ECM: Extracellular matrix
HVGs: High variable genes
DEGs: Differential expression genes
LV: Left ventricle
CFs: Cardiac fibroblasts
EMT: Epithelial-mesenchymal transition
