# Supplementary figures and images for: Understanding of the characteristics of fibroblasts in ischemic cardiomyopathy using single-nucleus RNA sequencing
Source: Sci Rep. 2025 May 30;15:18964. doi: 10.1038/s41598-025-00260-7 (PMC12125171; doi:10.1038/s41598-025-00260-7)

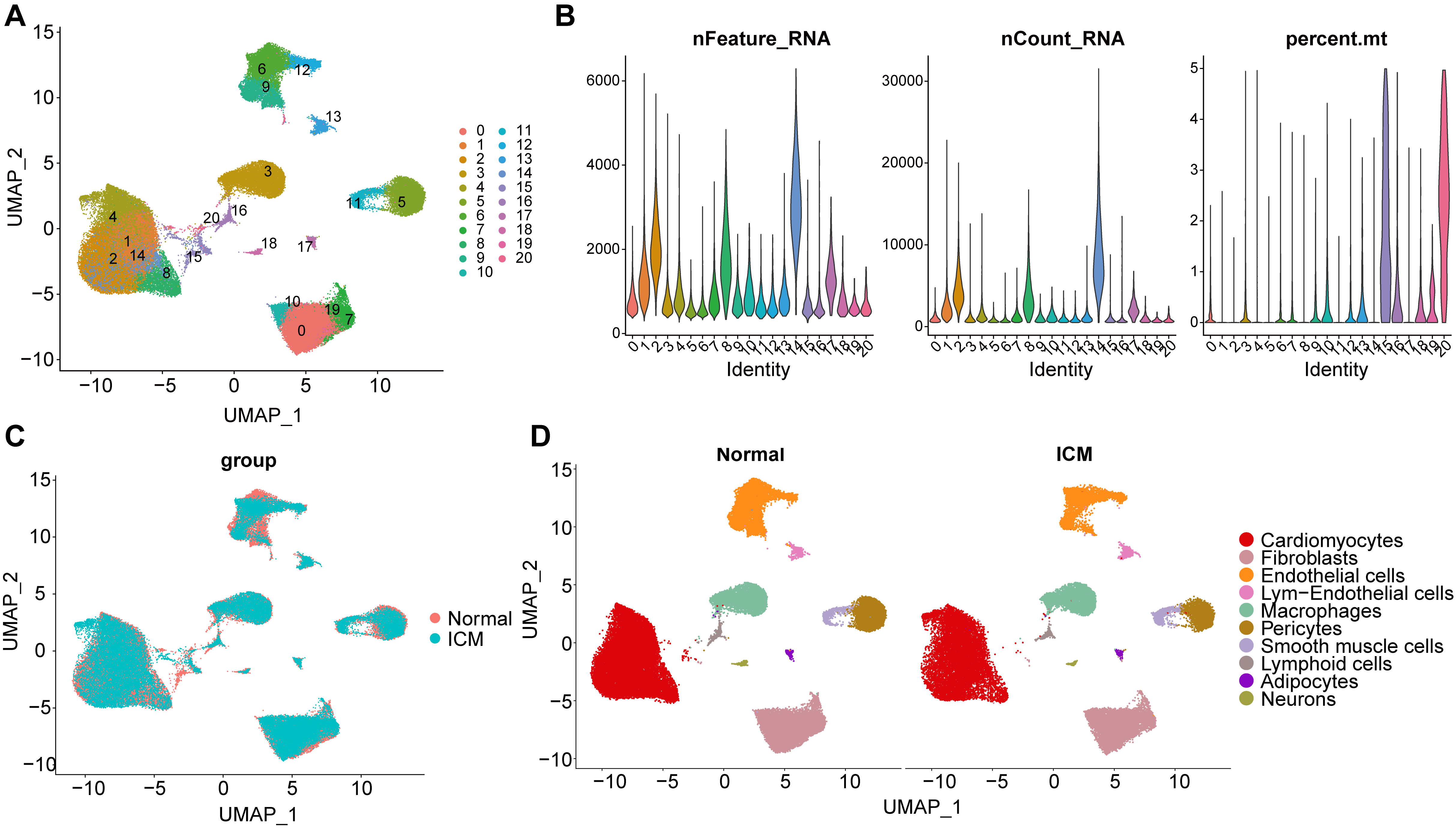

Supplement: Supplementary file 1 — Supplementary Material 1 [file 41598_2025_260_MOESM1_ESM.tif]

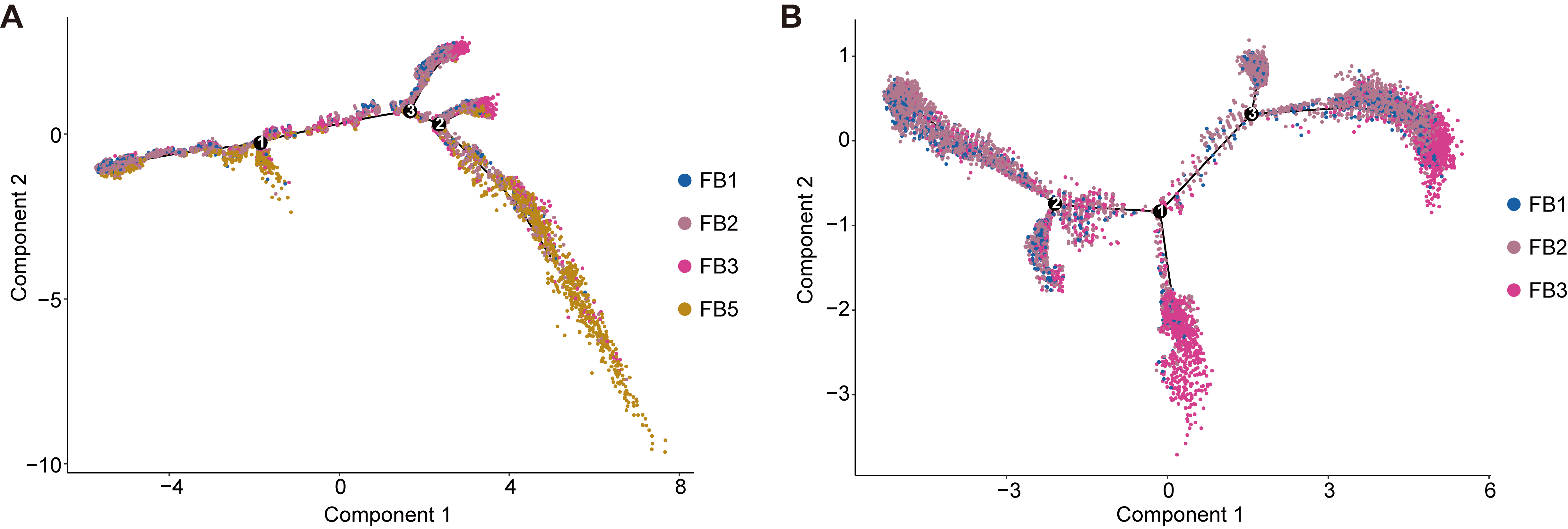

Supplement: Supplementary file 2 — Supplementary Material 2 [file 41598_2025_260_MOESM2_ESM.tif]

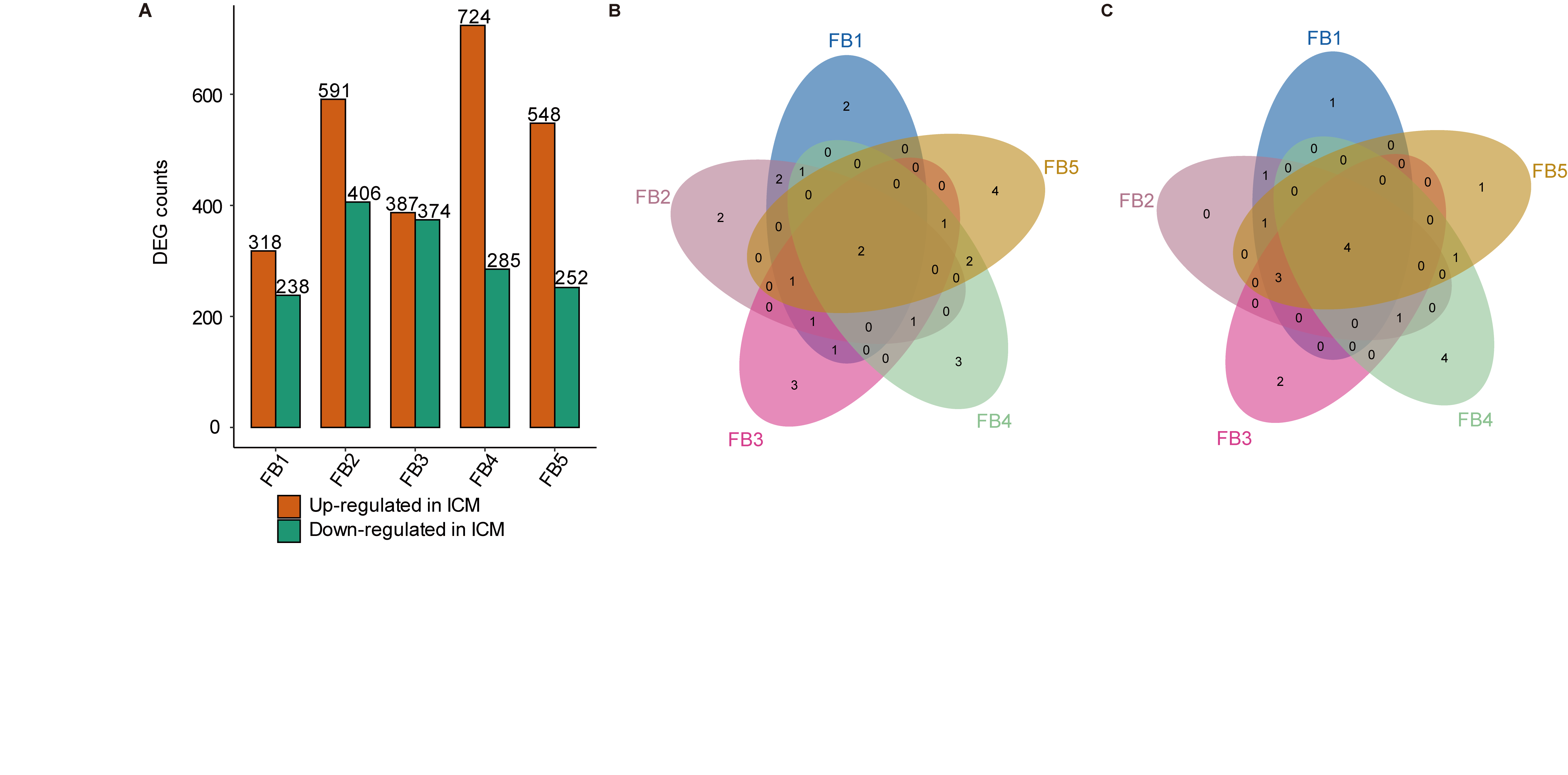

Supplement: Supplementary file 3 — Supplementary Material 3 [file 41598_2025_260_MOESM3_ESM.tif]

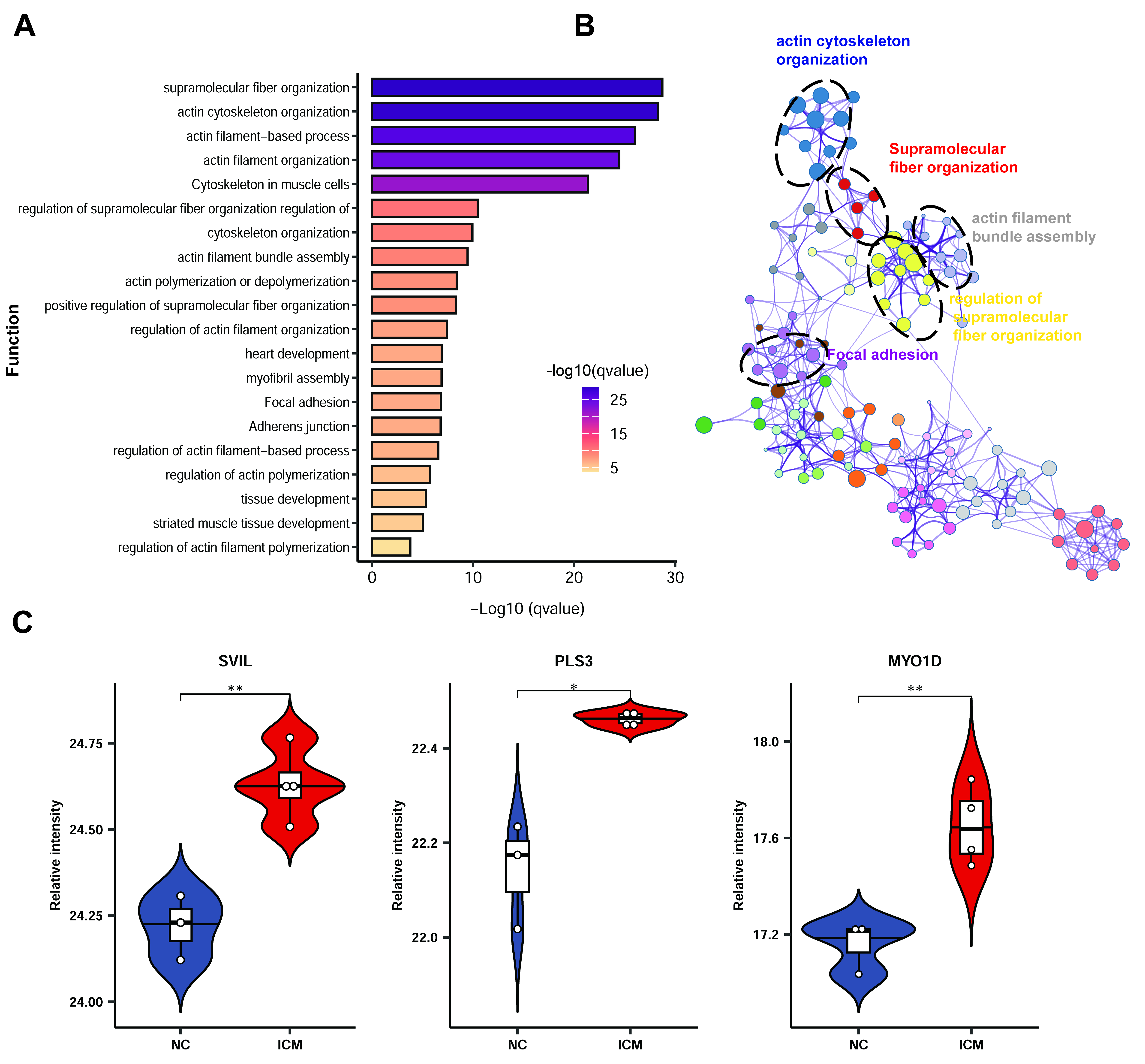

Supplement: Supplementary file 4 — Supplementary Material 4 [file 41598_2025_260_MOESM4_ESM.tif]

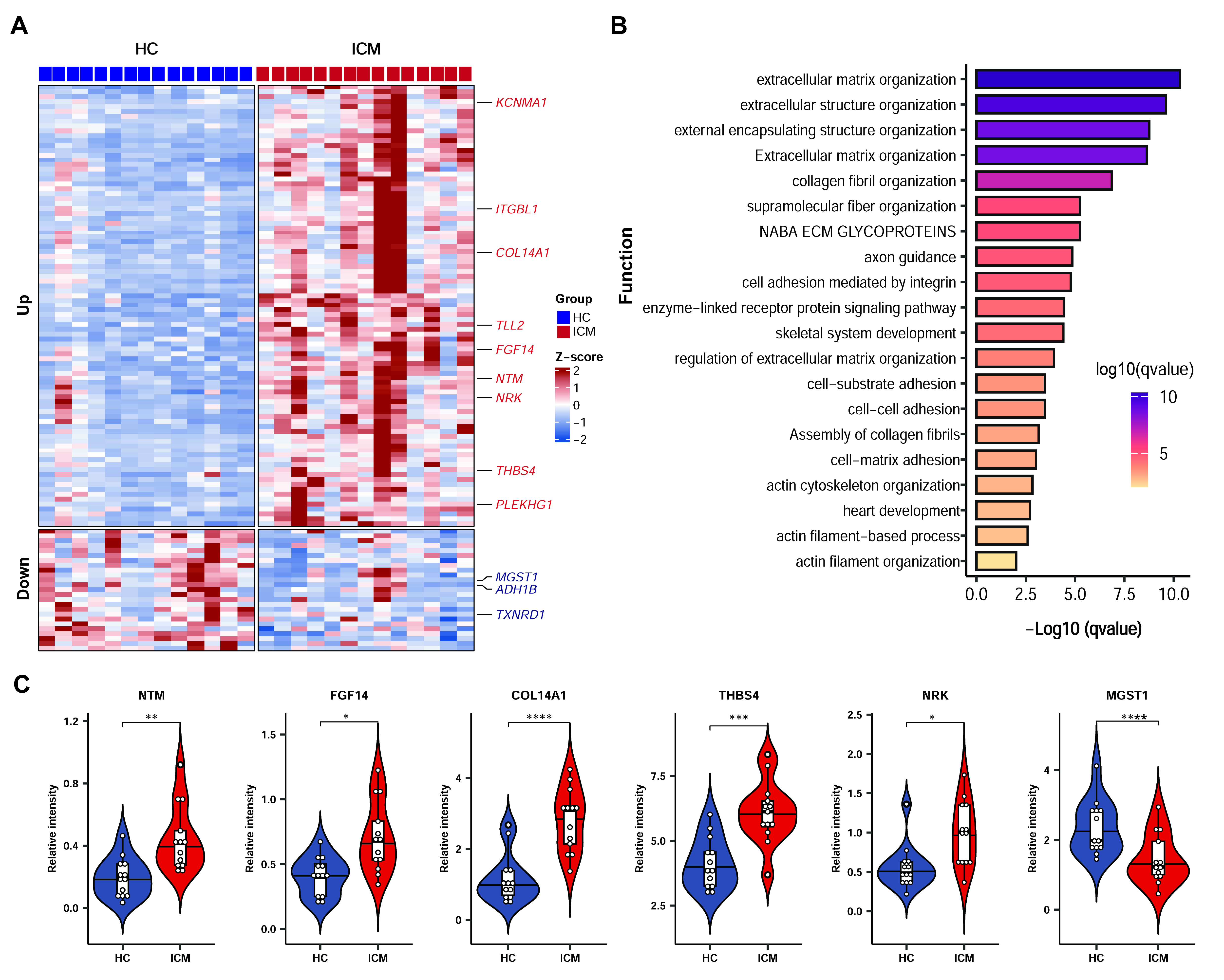

Supplement: Supplementary file 5 — Supplementary Material 5 [file 41598_2025_260_MOESM5_ESM.tif]

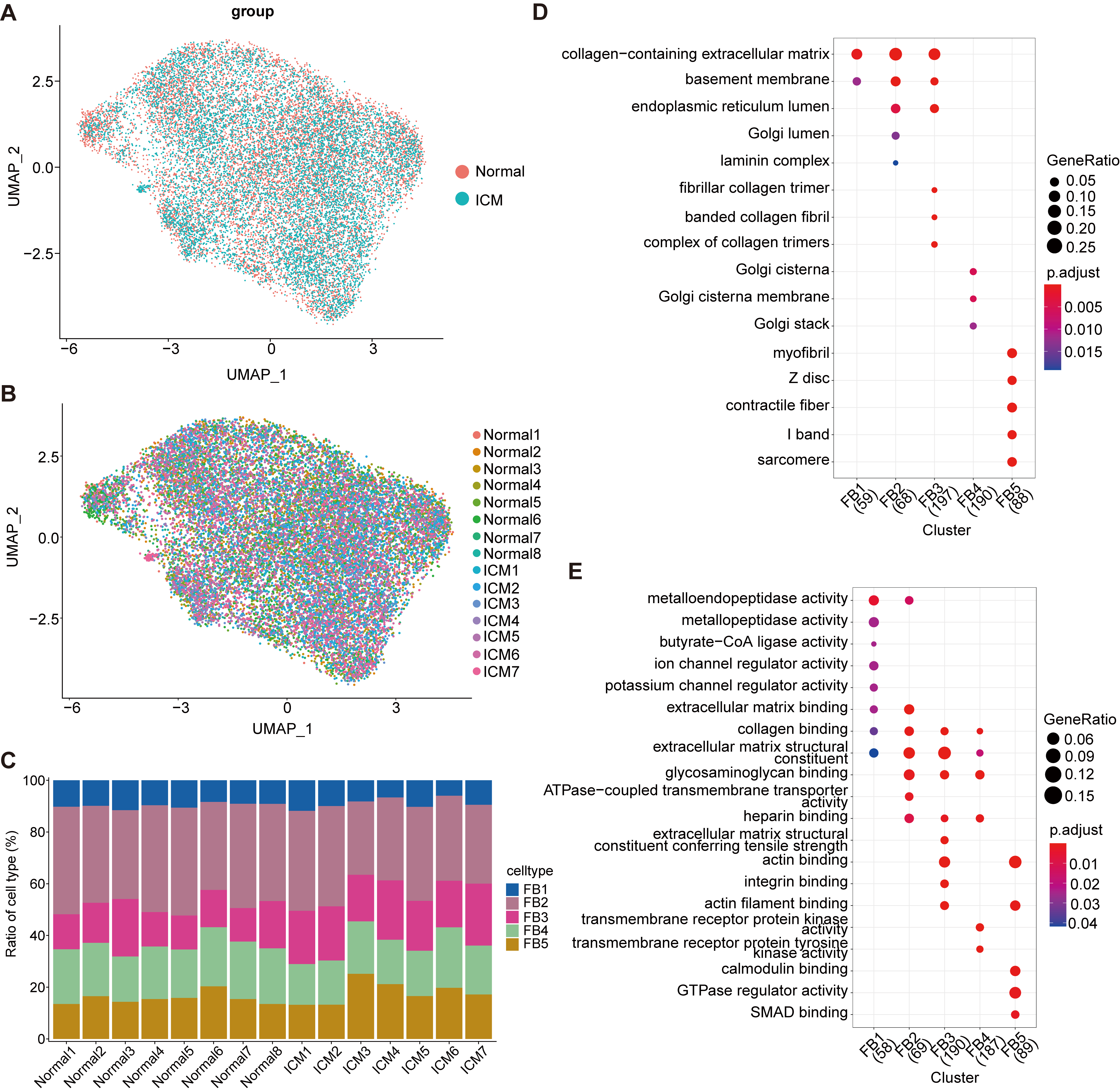

Supplement: Supplementary file 6 — Supplementary Material 6 [file 41598_2025_260_MOESM6_ESM.tif]
