# Supplementary material for: Understanding of the characteristics of fibroblasts in ischemic cardiomyopathy using single-nucleus RNA sequencing
Source: Sci Rep. 2025 May 30;15:18964. doi: 10.1038/s41598-025-00260-7 (PMC12125171; doi:10.1038/s41598-025-00260-7)
Supplement: Supplementary file 8 — Supplementary Material 8 [file 41598_2025_260_MOESM8_ESM.docx]

**
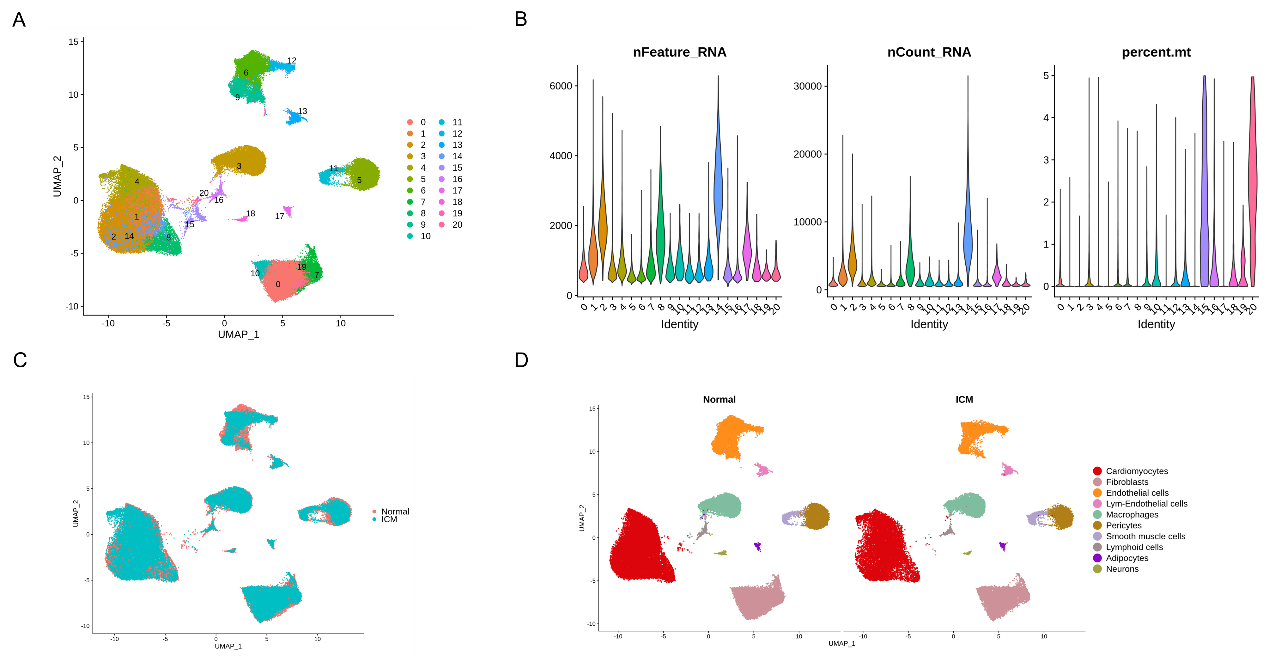
**

**FigS1. Unsupervised clustering of the ICM and Normal heart cells and the QC matrix.** (A) The landscape of unsupervised cell clustering, and shown using Uniform Manifold Approximation and Projection (UMAP) 2D dimensions. (B) The QC matrix focuses on nFeature_RNA (meaning the detected gene count for each cell), nCount_RNA (meaning the detected transcript count for each cell), and percent.mt (meaning the percent of transcript counts belong to mitochondrial genes) for each cluster. (C) The cell type UMPA plot is colored by group information. (D) The UMAP plot of cell types shows by splitted into Normal and ICM.


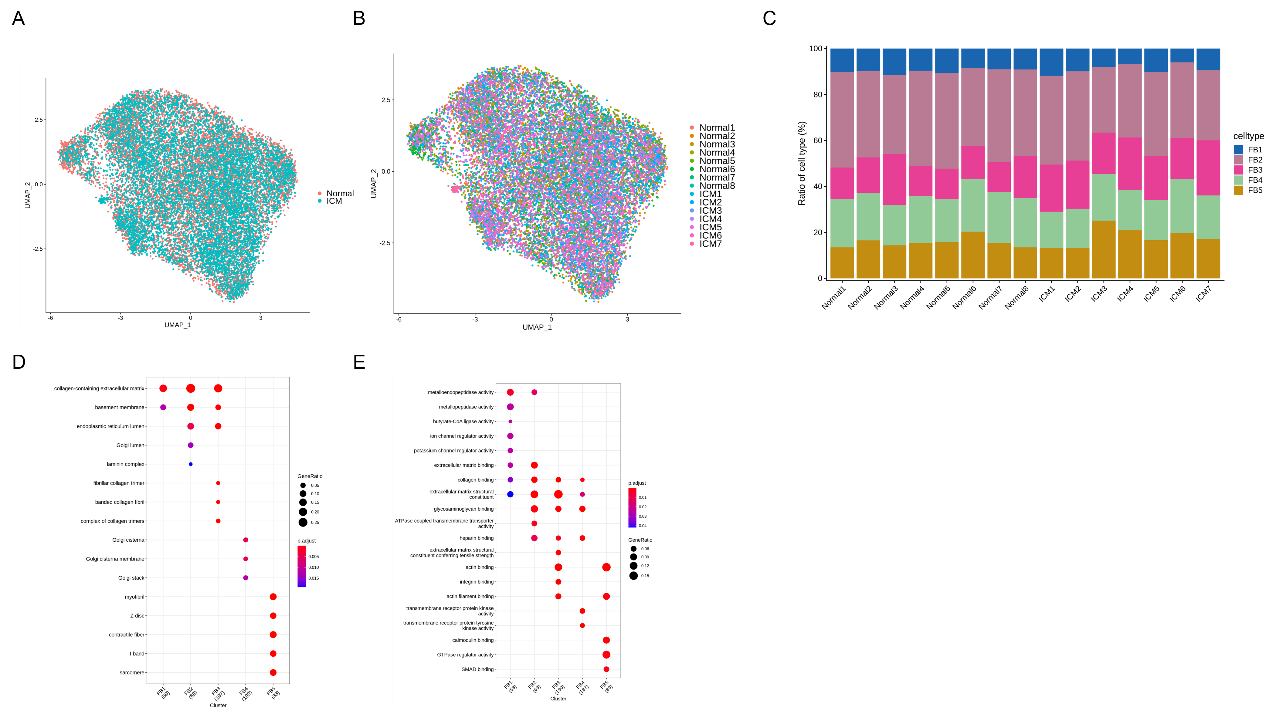


**FigS2. Unsupervised clustering of fibroblasts and the characteristics of the subtypes.** (A) UMAP plots of fibroblast subtype colored by group information. (B) UMAP plots of fibroblast subtype colored by sample information. (C) The cell type ratio of the fibroblast subtypes among different samples. (D) The GO enrichments in CC category for the marker genes of each fibroblast subtype. (E) The GO enrichments in MF category for the marker genes of each fibroblast subtype.

**
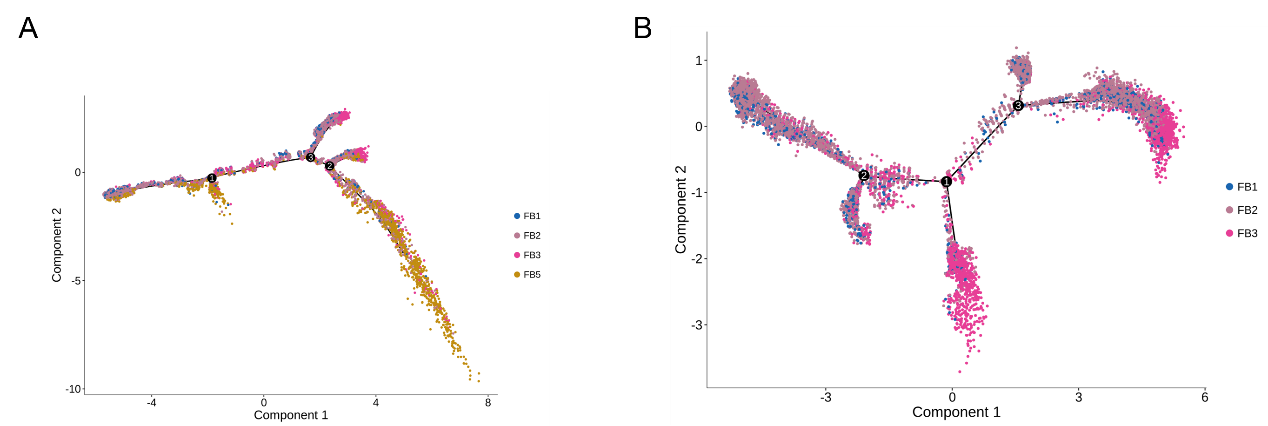
**

**FigS3. Differentiation trajectory under different fibroblast subtypes set.** (A) Differentiation trajectory of 4 cells come from FB1, FB2, FB3 and FB5, and the cellular points were colored by subtype information. (B) Differentiation trajectory of 3 fibroblast subtypes containing FB1, FB2, and FB3. The cellular points were colored by subtype information.


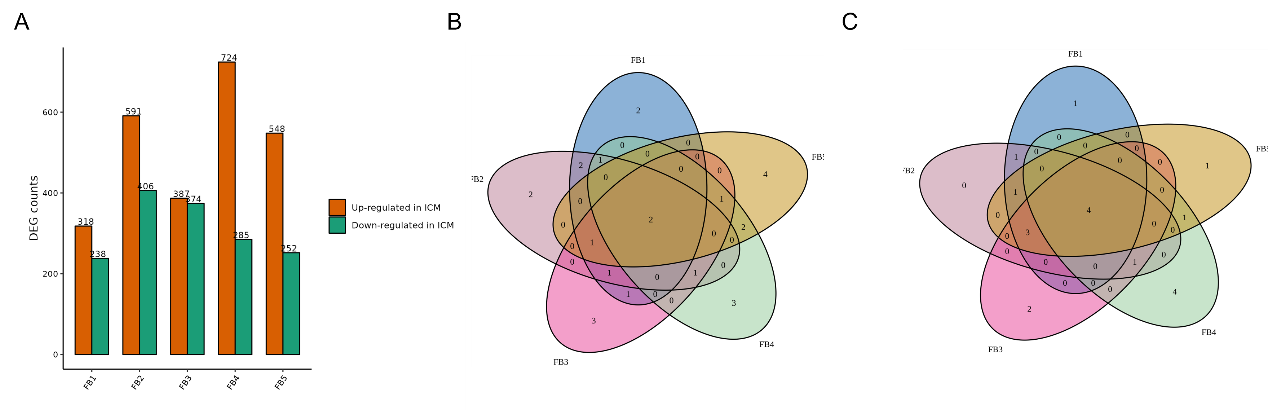


**FigS4. The expression of the DEGs between Normal and ICM among the fibroblast subtypes. (A)**The up-regulated and down-regulated gene counts in ICM compared to Normal for each fibroblast subtype separately. (B) Venn plot of the top 10 upregulated genes in ICM compared to Normal across the five fibroblast subtypes. (C) Venn plot of the top 10 downregulated genes in ICM compared to Normal across the five fibroblast subtypes.


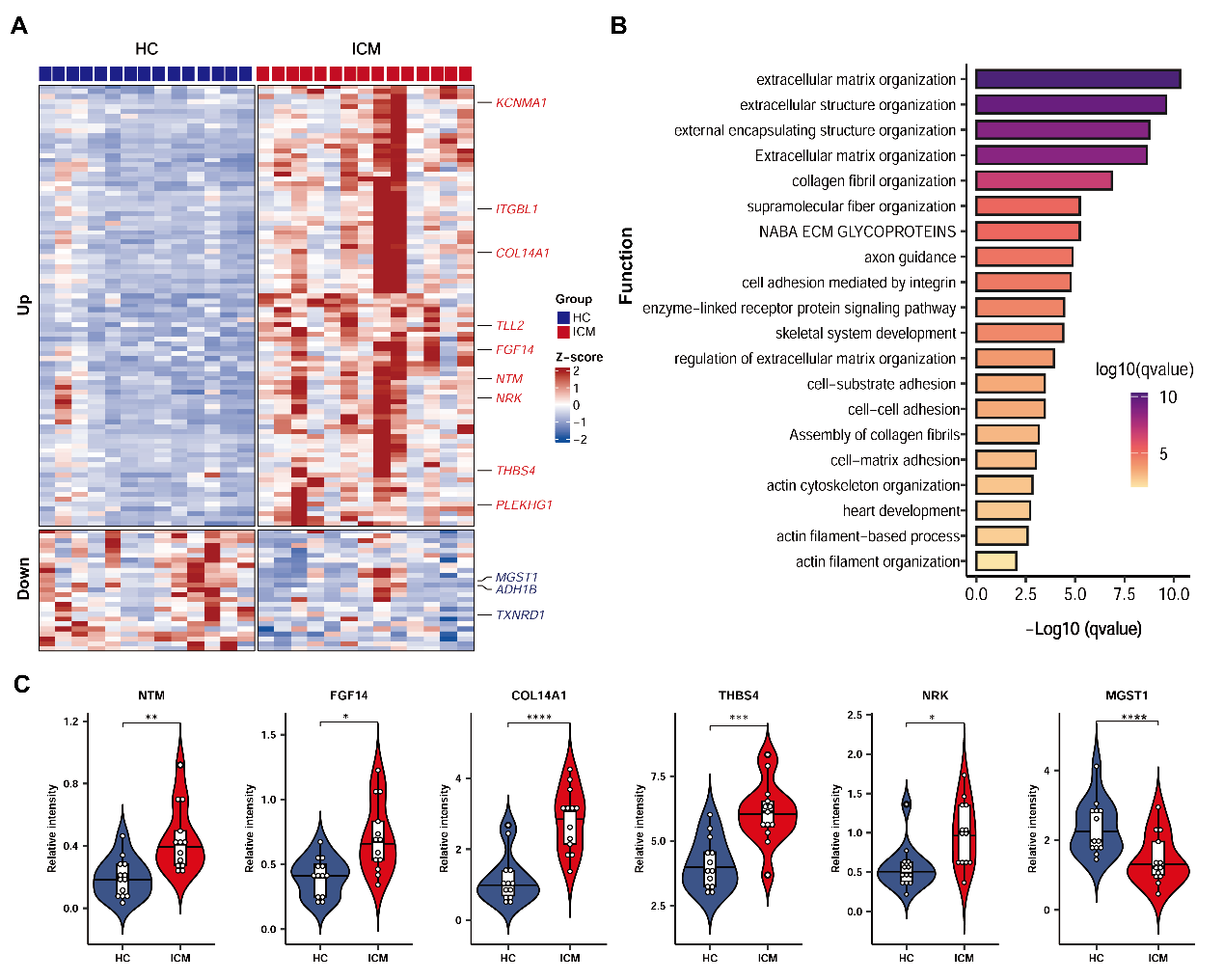


Figure S5. The differentially expressed genes (DEGs) identified in bulk RNA-seq data between HC and ICM samples

(A) The heatmap plot of the expression profiles of the DEGs, and the DEGs which overlapped with the top 20 up and down-regulated genes in FB3 were labeled. (B)The GO enrichments in BP category for the Up-regulated genes, and the top 5 terms focus on extracellular matrix organization, extracellular structure organization, external encapsulating structure organization, Extracellular matrix organization, collagen fibril organization. (C) Distribution of six differentially expressed genes, and NTM, THBS4, NRK, FGF14 and COL14A1 showed up-expressed in ICM, and MGST1 were down-expressed (* means padj < 0.05, ** means padj < 0.01, *** means padj <0.001, and **** means padj < 0.0001).


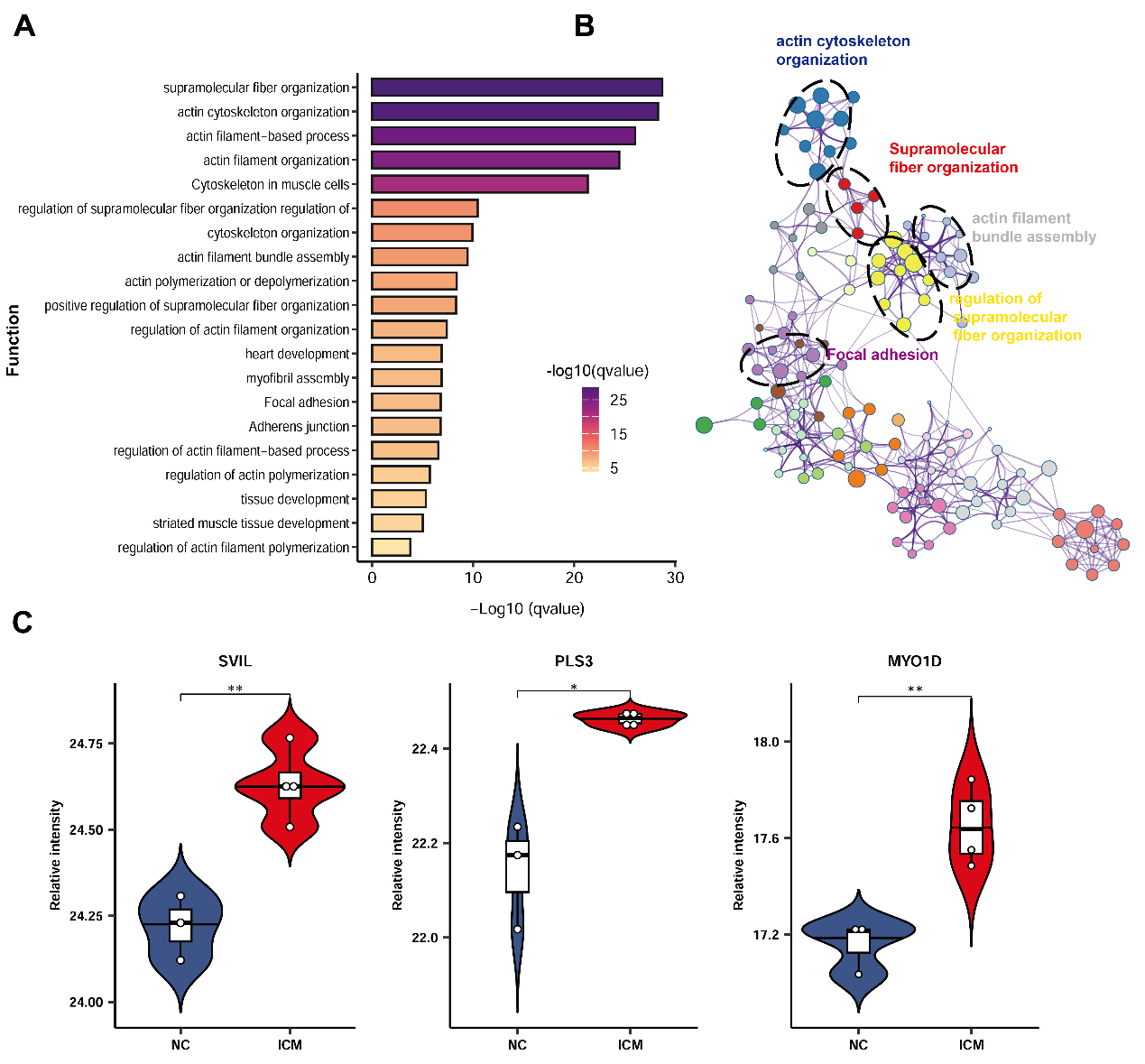


**Figure S6. Functional validation of external proteome data.** (A) Functional enrichment analysis of upregulated proteins in the ICM group reveals significant biological processes related to supramolecular fiber and actin cytoskeleton organization (higher -log10(q-value) indicates higher significance). (B) Functional interaction network analysis. (C) Distribution of differentially expressed proteins (p < 0.05 indicates a significant level, with statistical significance annotated based on student t test).
